# Predicting functions of maize proteins using graph convolutional network

**DOI:** 10.1186/s12859-020-03745-6

**Published:** 2020-12-16

**Authors:** Guangjie Zhou, Jun Wang, Xiangliang Zhang, Maozu Guo, Guoxian Yu

**Affiliations:** 1 School of Software, Shandong University, Jinan, China; 2 College of Computer and Information Sciences, Chongqing, China; 3 CEMSE, King Abdullah University of Science and Technology, Thuwal, Saudi Arabia; 4 School of Electrical and Information Engineering, Beijing University of Civil Engineering and Architecture, Beijing, China

**Keywords:** Gene ontology, GO terms, Maize, Protein function prediction, Graph convolutional network, Convolutional neural network

## Abstract

**Background:**

Maize (*Zea mays* ssp. mays L.) is the most widely grown and yield crop in the world, as well as an important model organism for fundamental research of the function of genes. The functions of Maize proteins are annotated using the Gene Ontology (GO), which has more than 40000 terms and organizes GO terms in a direct acyclic graph (DAG). It is a huge challenge to accurately annotate relevant GO terms to a Maize protein from such a large number of candidate GO terms. Some deep learning models have been proposed to predict the protein function, but the effectiveness of these approaches is unsatisfactory. One major reason is that they inadequately utilize the GO hierarchy.

**Results:**

To use the knowledge encoded in the GO hierarchy, we propose a deep Graph Convolutional Network (GCN) based model (DeepGOA) to predict GO annotations of proteins. DeepGOA firstly quantifies the correlations (or edges) between GO terms and updates the edge weights of the DAG by leveraging GO annotations and hierarchy, then learns the semantic representation and latent inter-relations of GO terms in the way by applying GCN on the updated DAG. Meanwhile, Convolutional Neural Network (CNN) is used to learn the feature representation of amino acid sequences with respect to the semantic representations. After that, DeepGOA computes the dot product of the two representations, which enable to train the whole network end-to-end coherently. Extensive experiments show that DeepGOA can effectively integrate GO structural information and amino acid information, and then annotates proteins accurately.

**Conclusions:**

Experiments on Maize PH207 inbred line and Human protein sequence dataset show that DeepGOA outperforms the state-of-the-art deep learning based methods. The ablation study proves that GCN can employ the knowledge of GO and boost the performance. Codes and datasets are available at http://mlda.swu.edu.cn/codes.php?name=DeepGOA.

## Background

Maize (*Zea mays* ssp. mays L.) has been subjected to cultivation and selection ever since over the past 10,000 years [1, 2]. Advances in sequencing technology have led to a large and rapidly increasing amount of Maize proteomic data (i.e., amino acid sequences and interaction networks). Knowledge of protein sequences is useful for many applications, such as yield and quality improvement, disease resistance and so on. Moreover, understanding the behavior of biological systems also requires determining the function of the protein [3, 4]. The functional annotations of proteins does not increase with the explosion of sequence data. Therefore, accurately annotating the functions of Maize proteins is crucial for all forms of basic and applied research [4–6]. However, due to the bias of botanists’ research interests, and identifying protein function always requires in vitro or in vivo experiments, only a very tiny part of newly obtained sequences have experimentally validated GO annotations [7–9]. Annotating proteins by wet-lab techniques (i.e., gene knockout and iRNA) is low-throughput and can not keep pace with the rapid influx of proteomic data. Therefore, the automatic methods have become increasingly important [4, 10].

**Fig. 1:**
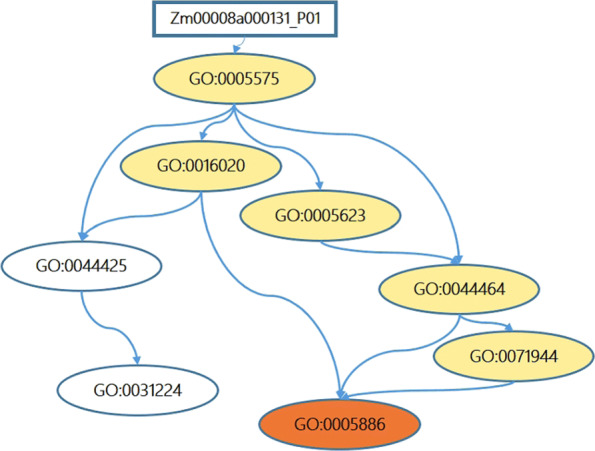
An example of hierarchical GO annotations of proteins. ‘Zm00008a000131-p01’ is a Maize protein, it is annotated with ‘GO:0005886’. According to the True Path Rule, the protein ‘Zm00008a000131-p01’ is also annotated with their ancestor terms (‘GO:0071944’, ‘GO:0044464’, ‘GO:0005623’, ‘GO:0016020’ and ‘GO:0005575’)

Gene Ontology (GO) is a controlled vocabulary of terms for describing the biological roles of genes and their products [11], it has been extensively used as a golden standard [12]. GO annotations of proteins are originally collected from published (or unpublished) experimental data by GO curators. GO includes plenty of GO terms and each GO term describes a distinct biological concept [13]. If a protein is annotated with a GO term, it means that the protein has the function represented by the GO term. Furthermore, many proteins do not only have a single function but may have multiple different functions, making the automated function prediction (AFP) become a multi-label problem. Additionally, the GO contains strong, formally defined relations between GO terms that need to be accounted during predicting the function of proteins. Till now, GO contains over 40000 terms, covering three different sub-ontologies, namely Biological Process(BP), Molecular Function (MF) and Cellular Component (CC). GO structurally organizes each sub-ontologies’ GO terms in a direct acyclic graph (DAG). In the DAG, each node corresponds to a GO term and each edge describes the relationship between terms. If a protein is annotated with a term, then the protein is also annotated with its ancestor (if any) terms. On the other hand, if a protein is not annotated with a GO term, the protein will not be annotated with any of its descendant terms. This rule is known as the True Path Rule [11, 14]: a child term is a further refinement of the function of its parental term. Figure 1 gives an example of GO annotations of Maize protein ‘Zm00008a000131-p01’.

A protein is typically annotated with multiple GO terms at the same time, since it usually participates in different life processes and executes multiple biological functions. The function of protein is not isolated. Multiple proteins form a biological pathway to implement biological functions, such as apoptosis and nerve impulses. Therefore, protein function prediction can be regarded as a multi-label learning problem [15–18]. However, due to a large amount of un-validated GO annotations of proteins, existing multi-label learning based function predicting methods face the issue of insufficient annotations and massive candidate GO terms. Furthermore, deep terms in the GO DAG describe more refined biological functions, and the shallow terms describe the broad functions. The missing GO annotations of proteins usually correspond to deep terms, which makes accurately predicting the GO annotations of proteins more difficult than traditional multi-label learning. Some efforts have been made toward utilizing the knowledge of GO. To name a few, Valentini [14] adjusts the predictions made by binary classifier for each GO term by using the GO hierarchy. Pandey et al. [19] firstly defined a taxonomic similarity through the knowledge of GO hierarchy, and used it to measure the correlations between GO terms, and then improved the prediction of deep GO terms via the correlation of GO terms. Yu et al. [18] views the GO structure as a graph and applied downward random walks (dRW) on the GO hierarchy. This method used the terms already annotated to the protein as the initial walkers to predict new GO annotation of this protein and identified the negative GO annotations of this protein [20]. Yu et al. [21] introduced a hybrid graph based on dRW, composed of two types of nodes (proteins and GO terms), to encode interactions between proteins, GO hierarchy and available annotations of proteins, and then predicted GO annotations of proteins through the bi-random walk algorithm proposed on the hybrid graph. Recently, Zhao et al. [22, 23] uses a hierarchy preserving hashing technique to keep the hierarchical order between GO terms and optimizes a series of hashing functions to encode massive GO terms via compact binary codes and then makes protein function prediction in the compressed hashing space and obtained a promising protein function prediction accuracy.

All the above methods can be regarded as shallow solutions, which are difficult to mine the deep (non-linear) relationship between proteins and GO terms. In recent years, deep learning has significantly sparked the development of image recognition and speech recognition [24]. The huge and complex output space is a big challenge faced by deep learning model in protein function prediction. Wehrmann et al. [25] established a series of fully connected neural networks for the GO terms of different levels in the GO hierarchy. They used each fully connected neural network as a classifier to predict a certain number of GO items separately. Since the frequency of GO terms annotated proteins on the same level also varies, which will impact the performance of the deep model, Zilke et al. [26] grouped GO terms based on the level of GO and the number of annotations. For each group, they established a fully connected neural network for the function prediction. Based on the fully connected neural network, Rifaioglu et al. [27] used conjoint triad [28], pseudo amino acid composition [29] and subsequence profile map [30] to obtain protein sequence features, which further improves the accuracy of protein prediction. These two deep learning based approaches separate GO terms, thus they can not well respect the connection between GO terms, which are not in the same group. Kulmanov et al. [31] first utilizes Convolutional Neural Networks to encode amino acids and incorporates the GO structure into the output layer. They generates a fully connected layer with a Sigmoid activation function for each GO term, which predicts whether the protein should be annotated with this GO term. Furthermore, they uses a maximum merge layer which outputs the maximum value of the classification results for all child nodes and the internal nodes to predict the non-leaf terms in the GO DAG. Kulmanov et al. [32] further removed the maximum merge layers and increased the number of convolution kernels to obtain a better prediction accuracy. These aforementioned deep models optimistically assume that their models are suitable for multiple GO terms. But in fact, they do not well utilize the hierarchical relationship between GO terms and still suffer from the gap between amino acids and GO annotations, which is often similarly termed as the semantic gap in image classification [33].

In this paper, We used the deep neural network to learn the knowledge of Gene Ontology and to reduce the semantic gaps between amino acids and Gene Ontology and the annotations. Particularly, the proposed DeepGOA extracts the feature vectors of amino acids using the Convolutional Neural Network (CNN), and learns the semantic representation of GO terms by the Graph Convolution Network (GCN) [34] referring to GO hierarchy and known annotations related with these GO terms. Then, DeepGOA learns a mapping from sequence features to the semantic space of GO terms. The mapping is learned by a multi-layer neural network, which is reversely guided by the known GO annotations of proteins. We observe that DeepGOA outperforms existing state-of-the-art methods [27, 31, 32, 35] on Maize PH207 inbred line and Human protein sequence dataset. In addition, DeepGOA retains more GO structure information. It is important to highlight that the deep learning model incorporating Gene Ontology structure, to the best of our knowledge, is still less studied in computational model-based protein function prediction. The conference and short version of DeepGOA [36], as a showcase of CNN and GCN for mining amino acids and Gene Ontology for protein function prediction, was published as part of IEEE International Conference on Bioinformatics and Biomedicine (BIBM 2019). In the extended version, we updated the background, problem definition, method description, results and their analysis.

## Results and discussion

In this section, we briefly introduce several widely-used protein function prediction evaluation criteria for performance comparison, and the recommended configuration of experiments. Then, we analyze and discuss the experimental results and compare our results with related and competitive approaches.

### Evaluation metrics

For a comprehensive evaluation, we use five widely-used evaluation metrics: AUC, AUPRC, PR50, *F*_*max*_ and *S*_*min*_ [37]. AUPRC (area under the precision-recall curve) and AUC (area under the receiver operator characteristics curve) are widely adopted for binary classification. Here we compute the AUPRC and AUC for each term and then take the average AUPRC and AUC of all terms. AUPRC is more sensitive to class-imbalance than AUC. PR50 is the average precision of all GO terms when the recall rate equals to 50%. *F*_*max*_ is the overall maximum harmonic mean of precision and recall across all possible thresholds on the predicted protein-term association matrix $\hat {Y}$. *S*_*min*_ uses the information theoretic analogs of precision and recall based on the GO hierarchy to measure the minimum semantic distance between the predictions and ground-truths across all possible thresholds. The first three evaluation metrics are term-centric ones and the last two are protein-centric ones. These metrics quantify the performance of protein function prediction from different perspectives and it is difficult for an approach to outperform another one consistently across all the metrics. It is worthwhile to point out that unlike other evaluation metric, the smaller the value of *S*_*min*_, the better the performance is.

*F*_*max*_ is a protein-centric F-measure computed over all prediction thresholds. First, we compute the average precision and recall using the following formulas:

1 $$ {p_{i}} = \frac{{\mathcal{T}{P_{i}}}}{{\mathcal{T}{P_{i}} + F{P_{i}}}}  $$

2 $$ {r_{i}} = \frac{{\mathcal{T}{P_{i}}}}{{\mathcal{T}{P_{i}} + F{N_{i}}}}  $$

3 $$ \Pr ecision = \frac{1}{N}{\sum\nolimits}_{i = 1}^{N} {{p_{i}}}  $$

4 $$ \text{Re} call = \frac{1}{N}{\sum\nolimits}_{i = 1}^{N} {{r_{i}}}  $$

We define $\mathcal {T}$ and *P* represent the protein’s true and predicted functions of proteins. $\mathcal {T}_{i}$ and *P*_*i*_ represent true and predicted values of the *i*-th protein functions. Where ${\mathcal {T}{P_{i}}}$ is the number of true positive, that is, the total number of occurrences of $\mathcal {T}_{i} = P_{i} = 1$. *FP*_*i*_ is the number of false positive, that is, the total number of occurrences of $\mathcal {T}_{i} =0$ but *P*_*i*_=1. *FN*_*i*_ is the number of false negative, that is the total number of occurrences of $\mathcal {T}_{i} =1$ but *P*_*i*_=0. *N* is the total number of proteins. Pr*ecision* and $\text {Re} call$ are so-called precision and recall, respectively. Then, we compute the *F*_*max*_ for all possible thresholds:

5 $$ {F_{\max }} = \underset{\theta \in [0,1]}{\max} \frac{{2p(\theta)r(\theta)}}{{p(\theta) + r(\theta)}}  $$

where $p(\theta) = \frac {1}{{m(\theta)}}{\sum \nolimits }_{i = 1}^{m(\theta)} {{p_{i}}(\theta)}$, *m*(*θ*) is the number of proteins whose predicted probability of at least one function tag is greater than or equal to the threshold *θ*, indicating the average precision of *m*(*θ*) proteins at the threshold *θ*. $r(\theta) = \frac {1}{{m(\theta)}}{\sum \nolimits }_{i = 1}^{m(\theta)}, {{r_{i}}(\theta)}$ is the average recall of all proteins at the threshold *θ*.

*S*_*min*_ computes semantic distance between real and predicted annotations based on information content of the classes. The information content *IC*(*t*) is computed by Eq. (14). *S*_*min*_ is computed utilizing the following formulas:

6 $$ {S_{\min }} = \underset{\theta \in [0,1]}{\min} \sqrt {ru{{(\theta)}^{2}} + mi{{(\theta)}^{2}}}  $$

7 $$ ru(\theta) = \frac{1}{N}{\sum\nolimits}_{i = 1}^{N} {{\sum\nolimits}_{t \in {{{\mathcal{P}}}_{i}}(\theta) - {{\mathcal{T}}_{i}}} {IC(t)} }  $$

8 $$ mi(\theta) = \frac{1}{N}{\sum\nolimits}_{i = 1}^{N} {{\sum\nolimits}_{t \in {{\mathcal{T}}_{i}} - {{\mathcal{P}}_{i}}(\theta)} {IC(t)} }  $$

where ${{{\mathcal {P}}_{i}}(\theta)}$ denotes a set of function labels whose prediction probability is greater than or equal to *θ*. ${{{\mathcal {T}}_{i}}}$ is a set of true annotations.

### Experimental setup

Our approach is implemented on Pytorch platform https://pytorch.org/. We conduct experiment on GO annotations and amino acids of Maize and Human. We firstly sort GO terms in descending order based on the number of proteins annotated to the GO term. Then we selected the most frequent terms for our experiments. Particularly, we select 117, 251 and 112 GO terms in BP, CC and MF for experiments on Maize; and 1190, 661 and 540 GO terms in BP, MF and CC for experiments on Human. After that, we use the information content of each GO term and the frequency of terms annotations to convert these selected GO terms into terms matrices and adjacency matrices. Meanwhile, we firstly convert each amino acid into a one-hot encoding and use a combination of one-hot vectors to represent the protein sequence. After that, we train CNN and GCN with graphisc processing unit (GPU). Finally, we fuse these two networks to predict association probabilities, and train these networks through the annotation information of the training protein sequences. In the following experiment, we randomly partition the proteins into a training set (80%) and a validation set (20%). All the experiments are performed on a server with following configurations:CentOS 7.3, 256GB RAM, Intel Exon E5-2678 v3 and NVIDIA Corporation GK110BGL [Tesla K40s].

### Results of protein function prediction

For experimental validation, we compare DeepGOA against Naive [4, 10], BLAST [35], Deepred [27], DeepGO [31] and DeepGOPlus [32]. Naive assigns the same GO terms to all proteins based on annotation frequencies. The idea of BLAST is to find similar sequeneces from the training data and transfer GO terms from the most similar. All input parameters are the same as those reported by authors or optimized within the recommended ranges. Since DeepGOPlus has too many parameters to run in our experimental environment, we reduce the number of convolution kernels from 512 to 128. Table 1 reveals the prediction results of DeepGOA and those of comparing methods in 10 rounds of independent partitions.

**Table 1:** Experimental results of predicting GO annotations of Maize and Human genome

		Maize					Human				
		PR50	AUC	AUPRC	*S*_*min*_*↓*	*F*_*max*_	PR50	AUC	AUPRC	*S*_*min*_*↓*	*F*_*max*_
BP	DeepGOA	**82.44**	**89.56**	**70.18**	**0.9965**	**67.53**	**55.20**	**69.79**	**62.20**	**19.7772**	**38.52**
	DeepGOPlus	76.47	89.52	69.64	1.2193	59.61	53.61	68.74	60.75	20.0152	36.23
	DeepGO	64.41	85.39	62.91	1.2586	59.49	50.25	63.85	57.13	20.6061	32.71
	Deepred	67.39	84.95	63.02	1.3509	58.21	55.60	68.33	56.96	19.9538	38.07
	BLAST	32.61	71.77	28.96	1.1745	61.10	46.50	57.72	48.94	20.2695	33.92
	Navie	27.08	49.93	27.67	1.8957	29.32	51.94	49.98	56.61	20.4729	34.45
CC	DeepGOA	**96.33**	**87.73**	75.78	**0.6603**	**75.74**	**50.88**	75.69	**49.97**	**4.9029**	**62.92**
	DeepGOPlus	91.21	82.51	**77.84**	0.8105	70.82	50.18	65.15	48.70	4.9488	62.75
	DeepGO	86.57	82.91	72.07	0.7759	71.08	43.60	69.51	44.81	5.1828	58.86
	Deepred	84.77	86.74	73.86	0.6952	69.85	44.58	**75.94**	44.58	5.8166	61.77
	BLAST	39.48	70.82	39.18	0.7904	62.02	21.25	56.27	26.91	5.0593	44.18
	Navie	48.14	49.98	43.74	1.2458	49.84	36.27	48.69	37.70	5.4474	55.15
MF	DeepGOA	**83.63**	**92.51**	**68.63**	1.7024	**58.10**	**68.64**	**82.03**	**70.98**	**4.7571**	**47.71**
	DeepGOPlus	72.70	83.67	64.42	**1.6777**	51.25	67.84	81.86	69.38	4.8426	46.82
	DeepGO	68.78	88.22	59.91	1.8551	52.82	54.56	75.98	62.47	5.2581	40.43
	Deepred	62.89	89.73	57.65	2.287	45.49	62.68	81.30	62.01	5.1711	45.14
	BLAST	27.40	67.76	32.92	1.8274	51.40	42.33	62.34	46.11	4.9195	41.07
	Navie	28.44	51.04	28.84	2.7430	26.13	46.86	49.87	52.77	5.7466	32.59

The best results for each metric are in boldface

Among the five evaluation metrics, DeepGOA consistently achieves better performance than these methods. The improvement of DeepGOA to other comparing methods with respect to AUPRC and PR50 is more prominent, which shows that DeepGOA can achieve effectiveness in dealing with the imbalances of GO terms by introducing GO structure. Besides, the performance of DeepGOA on the Maize protein dataset is better than the human protein dataset, because the annotations of Maize protein is more sparse than the annotations of human protein. Through the introduction of GO structure, DeepGOA can achieve better performance on relatively sparse data compared to other methods. The semantic representation of the GO term helps to improve this effectiveness. DeepGO uses the structure between parent and child terms in the final output layer, but still falls behind DeepGOA, which shows that the GCN we choose for GO hierarchy representation learning is more effective. DeepGOPlus does not use any GO structural information, but it gains better performance than DeepGO. This fact suggests that the structural regularization in the final layer of DeepGO does not make full use of the GO hierarchy. The performance margin between DeepGOA and DeepGOPlus again indicates the effectiveness of our coherent learning on the semantic representation of GO terms and the feature representations of amino acids. Deepred does not use the convolutional structure to learn the local features of the sequence but uses the fully connected layer to learn the protein sequence. Due to the sparseness of protein annotations, there are many false-negative predictions in this method, resulting in a higher AUC, but it does not perform well in AUPRC. The AUC value of Naive is always lower than 0.5, since it predicts the GO annotation of a protein based on the frequency of GO terms, and tends to assign the most frequent GO terms to a protein. Mostly, BLAST is inferior to other comparing methods (except Naive). This fact proves the effectiveness of learning the representation of amino acids by CNN for protein function prediction.

We choose one protein (Name:Zm00008a011322-p01) from our Maize protein dataset to illustrate the effectiveness of DeepGOA in the CC sub-ontology. Table 2 lists the GO annotations predicted by DeepGOA and other deep learning competing methods. The real annotation have been supplemented by True Path Rule. DeepGO annotates a GO term to a protein and automatically annotates all ancestor terms of that term to the protein simultaneously, due to the maximum merge layers. But the maximum merge layers of DeepGO will increase the false positive rate of the model. Compared with DeepGO, DeepGOplus uses a more reasonable convolutional structure and can mine deep terms. However, this method can not achieve the expected performance on the strong correlated GO terms because it ignores GO structural information. Deepred attempts to learn the overall features of the sequence based on a fully connected network, which leads to a situation that many annotations cannot be predicted. These results again confirms that DeepGOA performs better than other compared methods.

**Table 2:** The prediction of the Maize protein (Zm00008a011322-p01) with different methods

	Real annotation	DeepGOA	DeepGOplus	DeepGO	Deepred
CC	GO:0005622	GO:0005622	GO:0005622	GO:0005622	GO:0005622
	GO:0044464	GO:0044464	GO:0044464	GO:0044464	GO:0044464
	GO:0005623	GO:0005623	GO:0005623	GO:0005623	GO:0005623
	GO:0044424	GO:0044424	GO:0044424	GO:0044424	
	GO:0043229	GO:0043229	GO:0005737	GO:0043229	
	GO:0005737	GO:0005737		GO:0005737	
	GO:0043231	GO:0043231		GO:0043231	
	GO:0043227	GO:0043227			
	GO:0005634				

### Component and hyper-parameters analysis

In order to investigate which component of DeepGOA contribute to the improved performance of DeepGOA, we introduc three variants: DeepGOA-GO only uses the GO hierarchy; DeepGOA-LABEL only uses the co-annotation patterns without GO hierarchy; DeepGOA-CNN directly uses the representation of amino acids and the dot product to make function prediction, without using the semantic representation of GO terms. Table 3 lists the results of DeepGOA and its three variants on Human genome. The experimental configuration is the same as in the previous section.

**Table 3:** Prediction results of DeepGOA and its variants

		AUC	AUPRC	*S*_*min*_*↓*	*F*_*max*_
BP	DeepGOA	69.79	**62.20**	**19.7772**	**38.52**
	DeepGOA-GO	69.72	60.69	20.1579	36.79
	DeepGOA-Label	**70.12**	61.72	20.2206	38.14
	DeepGOA-CNN	69.19	61.06	20.2332	36.12
CC	DeepGOA	75.69	49.97	**4.9029**	**62.92**
	DeepGOA-GO	75.94	48.64	4.9127	62.43
	DeepGOA-Label	**76.83**	**55.87**	4.9707	62.67
	DeepGOA-CNN	74.85	49.19	5.0134	61.43
MF	DeepGOA	**82.03**	**70.98**	**4.7571**	**47.71**
	DeepGOA-GO	81.75	70.28	4.8201	46.98
	DeepGOA-Label	81.46	70.81	4.9661	46.88
	DeepGOA-CNN	77.65	63.12	5.2867	41.54

The best results for each metric are in boldface

DeepGOA generally has a better performance than its three variants due to the contribution of more valid information. Under the same experimental setting, DeepGOA-GO and DeepGOA-Label have better performance than DeepGOA-CNN. This observation proves that it is important and beneficial to learn the semantic representation of GO terms and optimize the mapping of feature representation of amino acids to the semantic representation. DeepGOA-GO achieves better results than DeepGOA-Label with respect to *S*_*min*_, since it utilizes the GO hierarchy while DeepGOA-Label mainly uses the co-annotation pattern of GO terms to the same proteins, and *S*_*min*_ is defined with respect to the GO hierarchy. On the other hand, DeepGOA-Label has better results on AUPRC and AUC by modeling GO term co-annotation. DeepGOA leverages the GO hierarchy and GO terms’ co-annotation pattern, and thus it obtains better results than three variants. This ablation study further confirms the necessity of incorporating GCN for exploring and exploiting the latent hierarchical relationship between GO terms, and thus to improve the prediction accuracy.

**Fig. 2:**
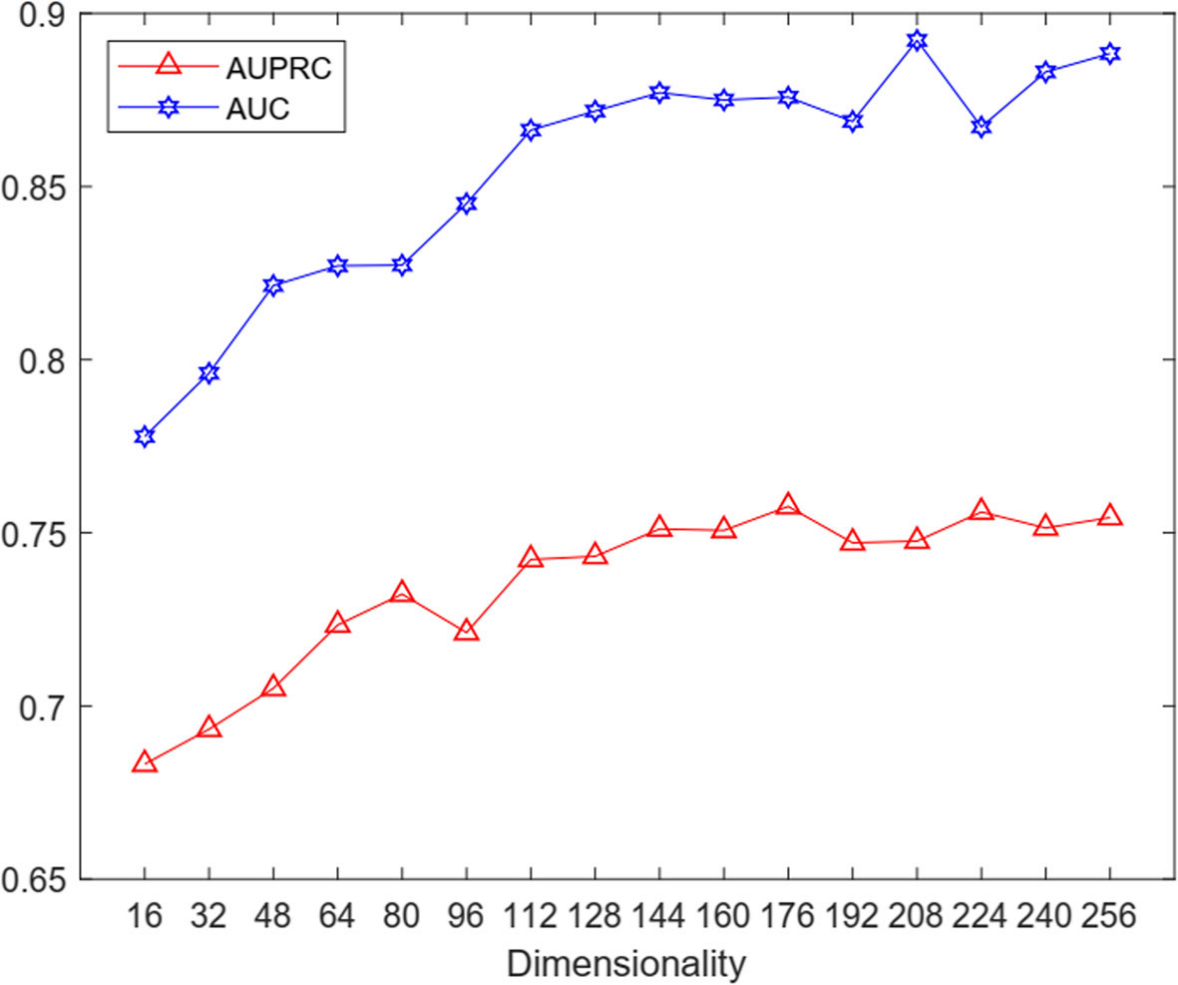
The AUC and AUPRC under different values of low-dimensional vector dimension

DeepGOA gives the predicted association probabilities by the dot product of the low-dimensional representation of the amino acid sequences and the low-dimensional representation of GO terms. If the dimensionality of low-dimensional representation is too low, it will lead to the loss of effective information. On the other hand, if it is too high, it will generate many parameters to degrade the training efficiency. Figure 2 reveals that when the low-dimensional vector dimension increases from 16 to 256, the AUPRC and AUC of DeepGOA prediction results will accordingly increase until stabilizing in the CC sub-ontology of Maize data. In our experiment, in order to make the experiment adapt to more GO terms and avoid the waste of computing resources, we chose 128 as the low-dimensional vector dimension.

## Conclusions and future work

Protein function prediction is one of the fundamental challenges in the post-genomic era. The firmly and formally defined relationship between the functions contained in the GO structure can improve the prediction performance. To this end, we develop DeepGOA based on GCN and CNN. DeepGOA utilizes the GCN to learn the semantic representation of GO terms through GO hierarchy and annotations related to GO terms, and the CNN to learn the representation of amino acids by combining the long and short range features of amino acid sequences. Then DeepGOA jointly seeks the mapping from the amino acids feature representation to GO terms semantic representation, and complete protein function prediction in an end-to-end and coherent manner. Experimental results on archived GO annotations dataset of Maize and Human show that DeepGOA outperforms existing deep learning-based protein function predicting models. Our ablation study further confirms that it is beneficial to learn the semantic representations of GO terms for function prediction. We will extend our work to predict the functional roles of diverse protein isoforms and noncoding RNAs.

## Methods

**Fig. 3:**
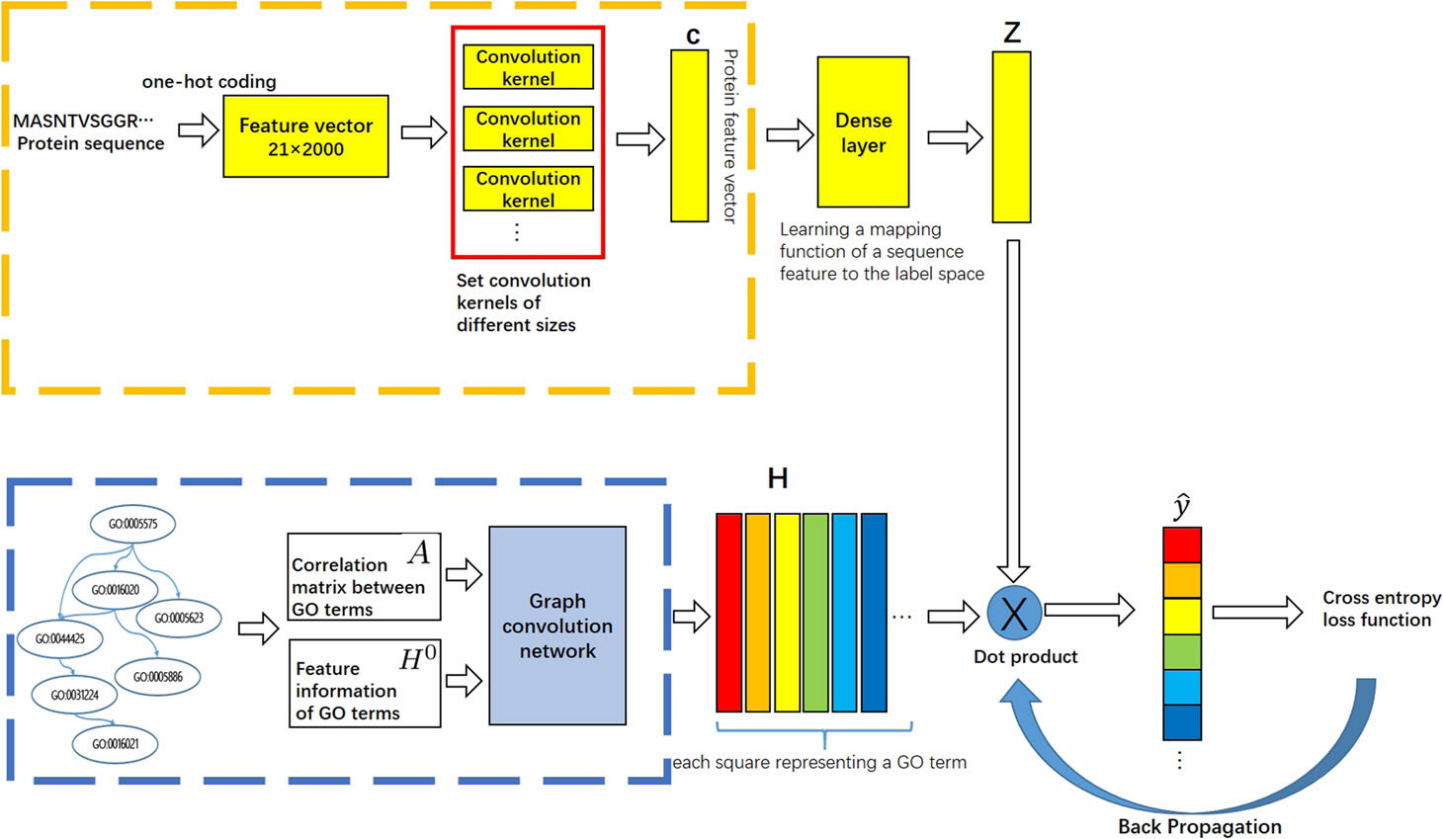
The network architecture of DeepGOA. The upper yellow subnetwork is the convolutional network part. The amino acids are extracted by convolution kernels of different sizes, and the fully connected layer is used to learn the mapping from sequence features to semantic representations of GO terms. The lower blue subnetwork is the graph convolution part, it uses the GO hierarchy ${H^{0}} \in {\mathbb {R}^{{\left | {\mathcal {T}} \right |} \times {\left | {\mathcal {T}} \right |}}}$ and empirical correlations between GO terms stored in $A \in {\mathbb {R}^{\left |\mathcal {T} \right | \times {\left | {\mathcal {T}} \right |}}}$ to learn the semantic representation of each GO term. The dot product is finally used to guide the mapping between proteins and GO terms and to reversely adjust the representations of proteins and GO terms. In this way, the associations between GO terms and proteins are also predicted

In the protein function prediction, effectively mining GO hierarchy and known annotation is important [12, 13, 22, 23]. The semantic and structural information of GO can largely assist computational models to determine the function of proteins. Recently, Deep learning has been widely used in the field of protein function prediction [25, 26, 31]. However, how to properly use the knowledge of GO in the deep model has been a huge challenge. Most deep models simply try to learn the mapping of protein sequences to GO terms directly, without respecting to the GO hierarchy when optimizing the mapping. Different from these methods, DeepGOA firstly learns the semantic representation of Gene Ontology via GCN and simultaneously optimizes the representation of protein sequence through CNN. After that, DeepGOA computes the dot product of the aforementioned two sub-nets to learn the mapping from feature representation to semantic representation in an end-to-end style. At the same time, it utilizes the collected annotations of proteins and back propagation to refine the mapping coefficients and to obtain coherent representations. Figure 3 illustrates the basic architecture of our model.

### Datasets

For our experiments, we downloaded the Gene Ontology data (June 2019) from GO official site^1^. GO data, which has three branches and 44,786 terms, includes 4169 terms in CC, 29,462 terms in BP, 11,155 terms in MF. We use Maize PH207 inbred line [38] sequence dataset to evaluate our approach. To prove the universality of our model, we also used the Human sequence protein dataset. We collect the protein sequence and GO annotation data of Maize PH207 inbred line from Phytozome^2^. The Maize PH207 inbred line protein data contains 18,533 protein sequences that annotated with one or more GO terms. We collected the reviewed and manually annotated protein sequences with GO annotations of Human from SwissProt,^3^ which contains 20,431 protein sequences.

For each subontology in GO, we all train a model to learn the knowledge of GO structure. Particularly, we rank GO terms by their number of annotations and select terms with the minimum number of annotations 25, 150 and 25 for CC, BP and MF, respectively. The adopted cutoff values are only half of those used by DeepGO [31], and thus our datasets include much more deep GO terms which describe more refined biological functions. Then, we propagate annotations by applying the True Path Rule. For instance, if a protein is annotated with a GO term, it will be annotated with all of its ancestor terms. We convert the annotations of protein into a binary label vector. If a protein sequence is annotated with a GO term from our list of selected terms, we will assign 1 to the term position in the binary vector and use it as a positive sample for this GO term. Otherwise, we will assign 0 and use it as a negative sample. In our model training process, we exclude proteins not annotated by any of the selected GO terms. In this paper, *n* represents the number of proteins in the training set, $\mathcal {T}$ represents the set of studied GO terms, $\left | {\mathcal {T}} \right |$ counts the number of selected GO terms.

### Extracting features from amino acids via CNN

Computers cannot directly identify amino acid sequences. Moreover, different proteins have different peptide chain structures and amino acid numbers. We need to numerically encode each amino acid sequence while retaining their characteristics. Kulmanov et al. [32] confirms that utilize one-hot encoding in deep networks can achieve a good predictive effect. Therefore, the input of our model is the one-hot encoding of amino acids. Each amino acid can be represented via a one-hot encoding vector of length of 21. There are twenty types of amino acids. Some amino acid sequences have undetermined amino acids at certain positions. We specifically use an additional one-hot bit to represent them. We transform each amino acid into a one-hot encoding and utilize a combination of one-hot vectors to represent the first-order structure of a protein. To ensure that the model input vectors are equal in length, we take the first 2000 amino acids for proteins vectors longer than 2000 amino acids and zero-padded for proteins vectors less than 2000 amino acids. We finally got the amino acid sequences feature vector with size 2000×21. Each amino acid sequence can be presented by a matrix:

9 $$ \boldsymbol{X}_{i}=[\boldsymbol{x}_{i1}, \boldsymbol{x}_{i2}, \ldots, \boldsymbol{x}_{i2000}]  $$

where $\boldsymbol {X}_{i} \in \mathbb {R}^{2000 \times 21}$ represents the *i*-th protein in the data set, **x**_*ij*_ is the one-hot encoding of the *j*-th amino acid of the *i*-th protein.

For each protein sequence feature vector, we utilize CNN to learn its low-dimensional representation. Convolutional Neural Networks (CNN) is a kind of feedforward neural network with convolutional computation and deep structure. It is one of the representative algorithms of deep learning and has a strong ability to extract features when processing fixed-size data. Therefore, we use a convolutional network to extract features from amino acid sequences and mine the deep information contained in the sequences. In addition, the amino acid sequence has not only a primary structure but also a secondary structure (*α*-helix and *β*-sheets) and a tertiary structure. This causes adjacent amino acids not necessarily participating in certain biological functions together. In order to dig out the impact of protein secondary and tertiary structure on function, we choose four different sizes of convolution kernels, respectively 8, 16, 24, 32, and set different sliding steps. The convolution portion takes **X** as input and extracts protein sequence features by a series of differently sized 1D convolution kernels. The convolution kernel is $\boldsymbol {w} \in \mathbb {R}^{21 \times h}$ and *h* is the sliding window length. The convolution operation is defined as follows:

10 $$ {\boldsymbol{c}_{im}} = f\left(\boldsymbol{w} * {x_{im:m + h}}\right),m \in [1,k - h]  $$

where ∗ is a convolution operation, ***w*** is a convolution kernel, *f*(·) is a non-linear operation, ***x*** is our model input vector, *k* is the input feature vector length. The new feature vector of ***c***_*i*_ is defined as:

11 $$ \boldsymbol{c}_{i} = \left[\boldsymbol{c}_{i1}, \boldsymbol{c}_{i2}, \ldots, \boldsymbol{c}_{ip}\right]  $$

where *p*=*k*−*h*+1. To this end, we get the feature representation of each protein.

Since our deep network has a lot of parameters and the loss function is used to optimize the training data, the neural network is very easy to get higher precision on the training data, but the poor results on the test data. Due to the unequal length of the protein sequence and the huge output space, it is easy to cause over-fitting. To solve this problem, We added two dropout layers in the fully connected layer of the convolution module. The role of the dropout layer is to stop the activation of a certain neuron with a certain probability *p* in forward propagation, which makes the model more generalized against relying too much on some local features. Protein function prediction is a multi-label learning problem and it is easy for the activation function to fall into the saturation region, causing the gradient disappearance. To solve this problem, a batch normalization layer is added after the convolution layer. The batch normalization layer aims to normalize the feature map generated by the convolution layer and leads parameters obeying the normal distribution.

### Graph convolutional network

Many existing protein function prediction methods utilize different techniques to employ the GO structure (or correlation) between terms and show improved per [21, 22, 31]. However, incorporating the GO structure into the deep model is a very challenging problem. For the learning of graph structure, traditional deep learning models can’t get a good performance, because they are designed for grids or simple sequences, such as images and texts. Graph Convolutional Network (GCN) [34] can learn the node representation of a graph (or network) using the graph structure. The core idea of GCN is to generate the representations of GO terms by propagating information between GO terms using the neighborhoods of GO terms. Unlike standard convolution for fixed-size input operations, GCN takes the feature descriptions ${\boldsymbol {H}^{0}} \in {\mathbb {R}^{{\left | {\mathcal {T}} \right |} \times {\left | {\mathcal {T}} \right |}}}$ with one-hot coding and the corresponding correlation matrix $\boldsymbol {A} \in {\mathbb {R}^{{\left | {\mathcal {T}} \right |} \times {\left | {\mathcal {T}} \right |}}}$ of GO terms as input, and updates the representation ${\boldsymbol {H}^{l}} \in {\mathbb {R}^{{\left | {\mathcal {T}} \right |} \times {d_{l}}}}$ of $\left | {\mathcal {T}} \right |$ GO terms. The operation of GCN layer is defined as follows:

12 $$ {\boldsymbol{H}^{l + 1}} = f\left({\hat{\boldsymbol{A}}}{\boldsymbol{H}^{l}}{\boldsymbol{W}^{l}}\right)  $$

where ${\hat {\boldsymbol {A}}} \in {\mathbb {R}^{{\left | {\mathcal {T}} \right |} \times {\left | {\mathcal {T}} \right |}}}$ is the normalized version of the correlation matrix ***A***, which will be given later. *f*(·) is a non-linear operation, and ${\boldsymbol {W}^{l}} \in {\mathbb {R}^{d_{l} \times d_{l+1}}}$ is a transformation matrix to be learned. We can learn the deep information of GO terms on the GO DAG by stacking the GCN layers.

The frequency of two terms annotated to the same protein is often used to estimate the correlation between GO terms, which has been widely adopted in multi-label learning based protein function prediction [15–17]. However, this simple estimation can not well reflect the underlying correlation between GO terms because the available annotations of proteins are imbalance and incomplete. Furthermore, the GO hierarchy between GO terms is independent from the known species. However, it has important guidance for accurate protein function, which is overlooked in this simple estimation process. In the Gene Ontology, the deep terms describe more refined biological functions. Therefore, the different information contents between GO terms are also the key information to estimate the correlation between GO terms. Given that, we combine the GO hierarchy and collected annotations of proteins to estimate the correlations between the parental term *t* and its child term *s* as follows

13 $$ A(t,s) = \frac{{{n_{s}}}}{{{n_{t}}}} + \frac{{IC(s)}}{{{\sum\nolimits}_{s' \in ch(t)} {IC\left(s'\right)} }}  $$

where *ch*(*t*) is an aggregation of all direct child terms of *t*, *n*_*s*_ and *n*_*t*_ represent the number of proteins annotated with term *s* and *t*, respectively. *IC*(*t*) is the information content of *t* and it is measured as:

14 $$  IC(t) = 1 - \frac{{\log (1 + \left| {desc(t)} \right|)}}{{\log \left| {\mathcal{T}} \right|}}  $$

where *desc*(*t*) includes all the descendants of *t* and itself. The semantic similarity between GO terms is widely measured utilizing this type of information content [20, 39, 40]. Obviously, since *t* has a lot of descendant GO terms, which convey more specific biological functions than *t*, the bigger the *desc*(*t*) is, the smaller the information content *t* has. This GO structure-based measurement is independent of the known GO annotations of proteins. Therefore, it is less affected by the incomplete and sparse GO annotations of proteins. In this way, we can differentiate the edges between parental terms and child terms.

### DeepGOA classifier learning

Till now, we can obtain the representation $\boldsymbol {H} \in {\mathbb {R}^{{\left | {\mathcal {T}} \right |}\times d}}$ for GO terms via the GCN, and the representation $\boldsymbol {Z}\in {\mathbb {R}^{n \times d}}$ of *n* protein sequences (after dense layer of *C* in Fig. 3) in the *d*-dimensional semantic space encoded by ***H***. Finally, we get the dot product of ***H*** and ***Z*** as the predicted association probabilities as follows:

15 $$ \hat{\boldsymbol{Y}} = \boldsymbol{H}\boldsymbol{Z}^{\mathrm{T}}  $$

Since it is a binary problem to predict the association between a GO term and a protein, and the semantic representation already encodes the latent relationships between GO terms, our multi-label loss function can be defined by cross-entropy as follows:

16 $$ Loss = \sum\limits_{s = 1}^{|\mathcal{T}|} {{y_{s}}\log \left(\sigma \left({{\hat y}_{s}}\right)\right)} + \left(1 - {y_{s}}\right)\log \left(1 - \sigma \left({{\hat y}_{s}}\right)\right)  $$

where $\mathbf {y} \in {\mathbb {R}^{\left | {\mathcal {T}} \right |}}$ stores the truth annotations of a protein, *y*_*s*_∈{0,1} denotes whether GO term *s* is annotated to the protein or not, *σ*(·) is the Sigmoid activation function.

By minimizing the above loss and back propagating the loss to the subnetwork of learning ***H*** and to the subnetwork of learning *Z*, we can achieve the optimization of ***H*** and ***Z***, and protein function prediction in the semantic space in a coherent end-to-end fashion.

## Data Availability

The source codes and datasets of DeepGOA are available at http://mlda.swu.edu.cn/codes.php?name=DeepGOA.

## Abbreviations

DeepGOA: a Deep learning framework for predicting gene ontology annotation
GO: Gene ontology
CNN: Convolutional neural network
GCN: Graph convolutional network
DAG: Direct acyclic graph

## Footnotes

1 http://geneontology.org/page/download-ontology

2 https://phytozome.jgi.doe.gov/pz/portal.html

3 http://www.uniprot.org/uniprot/
